# Effectiveness of Telemedicine and Teledentistry after the COVID-19 Pandemic

**DOI:** 10.3390/ijerph192113857

**Published:** 2022-10-25

**Authors:** Thomas Gerhard Wolf, Ralf Kurt Willy Schulze, Francisco Ramos-Gomez, Guglielmo Campus

**Affiliations:** 1 Department of Restorative, Preventive and Pediatric Dentistry, School of Dental Medicine, University of Bern, CH-3010 Bern, Switzerland; 2 Department of Periodontology and Operative Dentistry, University Medical Center of the Johannes Gutenberg-University Mainz, D-55131 Mainz, Germany; 3 Department of Oral Surgery and Stomatology, Division of Oral Diagnostic Sciences, University of Bern, CH-3010 Bern, Switzerland; 4 UCLA Center for Children’s Oral Health (UCCOH), UCLA School of Dentistry, Los Angeles, CA 90095-1668, USA; 5 Department of Surgery, Microsurgery and Medicine Sciences, School of Dentistry, University of Sassari, I-07100 Sassari, Italy

**Keywords:** application, digitalization, effectiveness, teledentistry, telemedicine, review

## Abstract

Telemedicine has become increasingly important worldwide over the last two decades. As a new field, it became known especially during the COVID-19 pandemic; this review presents fields of activity with special attention to opportunities and risks. Numerous areas of application offer the possibility for broad use in the medical and dental care landscape in diagnostics, therapy, rehabilitation, and decision advice across a spatial distance. Technical and semantic standards are required, and profiles and guidelines are increasingly defined and organized. Medical/dental consultations have been established in various regions around the world as a response to pandemic challenges and have made video and online emergency consultations possible. Telemedicine applications are already regularly used in medical/dental emergencies, regardless of the pandemic situation, both for transport by train and by plane, from which patients benefit. However, legal hurdles are often still unresolved, but infrastructure challenges both for provider, user hard- and software also complicate deployment. Problems are particularly prevalent in the absence of necessary internet coverage or among socially disadvantaged and vulnerable groups who cannot afford expensive equipment or do not know how to use the technology. Broad access must be enabled, and hardware and software interfaces and updates must be regularly checked and updated. Telemedicine might also improve access to and delivery of oral and general health care support both for rural and urban areas with low costs. Even though dentistry and many medical specialties are still performed clinically by means of practical/manual examination, there are areas of diagnostics where telemedicine applications can provide good support. Therefore, as conclusions, access, and delivery of telemedicine applications in dentistry and medicine should be expanded and improved to provide access to all population groups.

## 1. Introduction

Telemedicine methods are increasingly being used in patient care for a wide range of applications. The term telemedicine stands for the use of information and communication technologies to provide different medical or dental care concepts with the basic approach of providing medical services for (oral) health care in the areas of diagnostics, therapy, and rehabilitation, as well as decision-making advice by the physician over a spatial distance or possibly even a temporal offset [1]. In 2015, the working group telemedicine of the German Medical Association dealt with the definition of the term, as a great deal of ambiguity had become apparent in public usage. According to the World Health Organization [2], the term eHealth (electronic health) is the designation for the secure and cost-effective use of information and communication technologies. This use is aimed at promoting general health and includes not only health promotion and reporting but also health systems in general, general knowledge, and research. Accordingly, the following definition is proposed for telemedicine methods in health care by the working group telemedicine of the German Medical Association [3]:

“Telemedicine is a collective term for various medical care concepts, which have in common the principal approach that medical services of health care for the population in the areas of diagnostics, therapy, and rehabilitation, as well as in medical decision-making consultation are provided over spatial distances (or temporal offset). Information and communication technologies are used for this purpose.” [3].

Furthermore, the working group telemedicine recommends refraining from presenting a separate field of telemedicine since telemedical methods are an integral part of almost all medical or dental specialties. Therefore, the terminology of telemedical methods should be applied to the health care of the population.

This narrative review deals with the presentation of the fields of activity of telemedicine in hospitals and in the dental field, with special attention to opportunities and risks. The fields are examined from different perspectives of the stakeholders involved and critically reflected on and discussed.

## 2. Areas of Application of Telemedicine

**Figure 1:**
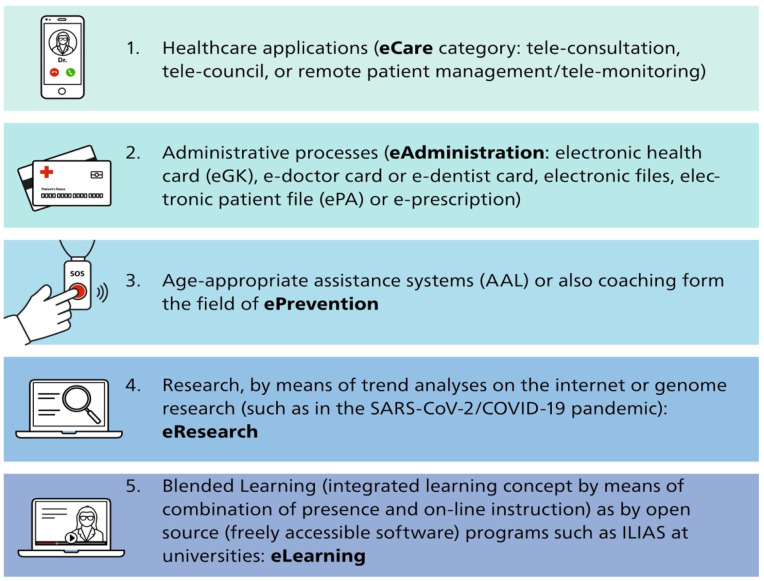
Telemedicine defined by the working group telemedicine of the German Medical Association [1,3].

The field of telemedicine must be defined more broadly in the context of eHealth discussions [3]. It can be divided into five areas (Figure 1). Each area offers numerous applications as well as further development areas, which can bring additional advantages for the respective users. An established field of telemedicine is teleradiology, which has been evolving over 50 years ago. Although the concept was developed even earlier, teleradiology essentially started with television connections within hospitals in the 1960s [4]. The American College of Radiology (ACR) defines teleradiology as the electronic transmission of radiologic images from one location to another for the purposes of interpretation and/or consultation [5]. Obviously, digital systems and image data fostered the application and usefulness of teleradiology.

## 3. Digital Infrastructure

### 3.1. Central Directory for Technical and Semantic Standards, Profiles, and Guidelines

The so-called ‘VeSta’ (Verzeichnis für informationstechnische Standards) interoperability directory of the German healthcare system consists of two parts: the ‘VeSta information portal’ and the online platform ‘VeSta standards.’ As an independent directory, the ‘VeSta information portal’ provides an overview of telemedicine projects and electronic applications in healthcare [6]. It is intended to provide more transparency and to avoid isolated solutions in Germany. It should also help identify hurdles to the integration of helpful telemedicine applications in everyday life, since in the past, telemedicine projects were frequently launched and often could not be integrated into standard care after the end of the funding period. ‘VeSta standards’ aims to list, evaluate, and recommend technical standards, profiles, and guidelines of information technology systems regarding financial implications. It is intended to serve as a central point of contact for a digital health system and to promote dialogue between stakeholders from science, politics, and providers of electronic applications [6].

### 3.2. Infrastructural and Technical Challenges

Especially in rural areas, there are still numerous areas that are not yet optimally covered by various mobile network providers (e.g., Telekom, Vodafone, O_2_, etc.) using cell towers. While the European Union (EU) improved the internal market in 2017 by enabling borderless telephony and internet surfing by eliminating roaming charges for citizens of EU member states as well as the European Economic Area (EEA), studies from 2015 and 2018 reveal glaring weaknesses. Europe continues to lag the U.S. at 15% and Asia, such as South Korea, at 70% in LTE connectivity [7]. According to this study, it was indeed expected that Western European countries would catch up and have an LTE market share of 85% by 2020. However, when considering the fourth-generation LTE mobile communications standard with transmission speeds of up to 300 megabits per second, further development of the new and even more flexible 5G mobile communications standard, which is expected to handle much larger volumes of data, must already be considered. A worldwide penetration of smartphones by 2020 was indicated in the Ericsson study, at 70% of the world’s population. In a comparison within the European Union, Germany was found to be one of the laggards, with only an average download rate of about 22.7 MB/s and a network coverage of 65.7% for the LTE network [7]. While countries such as the Netherlands, Norway, Hungary, Belgium, and Bulgaria only have download speeds between 42.1 and 33.3 MB/s, the availability of network coverage in these countries is even between 74.0% and 92.2% [8].

## 4. Impact of the SARS-CoV-2/COVID-19 Pandemic

The SARS-CoV-2/COVID-19 pandemic has caused a gross disruption in the daily lives of many people around the world and has had a lasting impact on the world. Through numerous measures, physical contact with other people, family, friends, and acquaintances, as well as professionally with colleagues and business partners, was largely restricted or even partially prohibited through a so-called lockdown. Worldwide, travel warnings were issued, non-essential travel was partially restricted, or even entry bans were issued by countries or made subject to conditions. In addition to measures such as quarantine or isolation, there were also numerous recreational and cultural venues, restaurants, and retail outlets temporarily closed, and in large parts of the world, mandatory surgical masks, or even FFP2 masks, were adopted by country governments. There were regular adjustments, most of them weekly, to the local, regional, or country-specific situation in consideration of various objective measures, such as the R-value, daily infection counts, hospital bed occupancy, or intensive care unit occupancy. The situation was regularly reviewed and reassessed [9]. Dental practices, and thus dental services, were also affected by the impact of the pandemic, both in Germany [10] and worldwide [11,12]. In Germany, elective (scheduled, deferrable) treatments were generally suspended by the legislature during the period of the initial lockdown in the spring of 2020 in several states. In some states, these procedures were to be postponed with an urgent recommendation. Only emergency treatments were exempted from this regulation nationwide, which logically led to an immediate reduction in working hours, as well as a treatment backlog for patients. While high-risk patients, for the most part, decided on their own to suspend or postpone dental treatments due to possible aerosol formation and contact with other persons, the displeasure of numerous patients increased with an increase in pain levels as the lockdown period progressed. Finally, the long-awaited crown and bridge restoration, or even a chronically painful tooth, could finally be extracted after the lockdown period. To ensure that the various parties involved in the oral health care process (e.g., dentists, dental technicians, general practitioners, specialists, midwives, speech therapists) as well as the health insurance funds and health insurers are kept informed, a joint package of measures was developed by the dentists’ representative body. The two German corporations under public law, the Kassenzahnärztliche Bundesvereinigung (KZBV) and Kassenzahnärztliche Vereinigungen der Länder (KZV) and the association of the Landeszahnärztekammern, organized as an association, the German Dental Association (BZÄK) were the initiators and opinion leaders of this package of measures, which was necessary to maintain oral health care in even as the SARS-CoV-2/COVID-19 pandemic continued to spread [13]. This coordinated effort at the federal and state levels, in addition to ensuring a synchronized level of information, significantly minimized, and ultimately prevented uncertainty among dentists and patients. To also ensure dental care for infected and quarantined patients, recommendations and suggestions were developed in compliance with the currently valid infection protection regulations, as well as offering specialized practices and treatment centers in hospitals or in affiliated oral and maxillofacial surgery clinics. Treatment of such infected or quarantined/isolated patients in normal dental practices should therefore be avoided to protect the general population. The risk of infection for patients and practice staff should be reduced, the spread of the virus during dental treatment should be prevented as far as possible, and care should also be safeguarded in the long term. Various dental practices had to close temporarily in exceptional cases with justified specificity, which was to be done in coordination with the local health department or the responsible Regional Dental Authority (KZV) in Germany. In the second year after the start of the pandemic (2021–2022), the situation in dental practices returned to normal, although many patients still wanted the number of visits reduced to a necessary level.

### Telemedicine/Teledentistry as a Response to Pandemic Challenges

The SARS-CoV-2/COVID-19 pandemic has given enormous impetus to the use of telemedicine services. Not only high-risk patients who were particularly at risk appreciated this kind of presentation to their general practitioner, specialist, and dentist. In this pandemic situation, the German Bundestag and the federal government took numerous measures and passed ordinances and laws that advanced the digitization of healthcare [14]. A law on digital care paved the way for digital applications in everyday practice. For this, it was necessary for the so-called ban on remote treatment for doctors or dentists to fall [15]. A consultation by video via cell phone or computer was taken up by numerous patients as the first point of contact, which was unthinkable just 25 years ago. Technological advancements and the proliferation of smart (cell) phones have contributed greatly to this. The path toward an electronic patient record has already been discussed for several years by decision makers in the healthcare sector, with both advantages and disadvantages.

## 5. Regular Use of Telemedicine Applications for Emergency Treatment & *Consilium*

### 5.1. Use of Telemedicine in Aviation

In recent years, an increase in medical incidents on board aircraft has been observed in global air traffic [16,17]. According to Lufthansa AG Group data (Cologne, Germany), between 10 and 15 medical incidents occur on approximately 1700 daily flights. These include asthma attacks, strokes, colic, or even suspected heart attacks, as well as minor illnesses with symptoms such as headaches, fever, or vomiting [18]. Various resources are available on board, such as a so-called Doctors’s Kit or a First Aid Kit, the latter of which may also be used by non-professional helpers. For some years now, the possibility of round-the-clock advice from InternationalSOS has been of particular importance at this point. InternationalSOS is a service provider that offers advice 24 h a day, seven days a week, with qualified flight and emergency physicians as medical advisors via satellite telephone, even on long-haul flights. The situation on board is characterized by limited space and challenges for the human body, such as time differences, reduced oxygen partial and ambient pressure, and air dryness. Demographic changes are also visible in air travel. There are increasingly older passengers, often with more pre-existing conditions [16,17]. Additionally, it must be considered that air travel is lengthening, as modern aircraft can travel longer distances. Therefore, as travel stress increases for passengers, an increase in emergencies in the aircraft is also expected. A medical emergency is expected to occur on board a passenger aircraft every 12 min worldwide, but this is comparatively very low, at eight to 50 per 1 million passengers. While the probability of an unscheduled landing is 1 in 1 million passengers, the probability of a fatality is 1: approximately 2 million passengers [19]. While cabin crews are trained/educated annually for anticipated emergencies up to and including cardiopulmonary resuscitation and the use of an automatic defibrillator (AED), they still require support from physicians for more serious emergencies. The situation on board the aircraft is special. Space conditions are cramped, and access to the patient is difficult. Shielding from other passengers is usually difficult or impossible. In addition, there are language problems and limited technical and thus also medical therapeutic possibilities. The limits of diagnosis are reached more quickly, and a simple blood pressure measurement, including auscultation, may not be possible due to ambient noise. At this point, telemedical consultation options come to the fore for exchange for both lay helpers and physicians in consilience with qualified colleagues on the ground [20]. While the pilot/flight captain of the aircraft is in close contact with a physician on board and consults, the *consilium* with a physician experienced in flight operational aspects is also an important support in such unplanned emergency situations; for example, to consult on points such as an evasive landing or intermediate landing [21]. While telemedicine was mentioned as early as 2003 with the creation of an internet option (“Flynet,” i.e., W-LAN on board the aircraft) together with the German Aerospace Center (DLR), medical devices were already tested for their practicality at that time. While internet telephony is often prohibited for passengers on flights, it should be possible to activate the technical option with sound and image transmission in an emergency. Qualified physicians from various clinics with different specialties can serve as emergency call centers. According to the airline [18], state-of-the-art medical technology is already in use on aircrafts today. For example, electrocardiography (ECG) data can be transmitted to centers of expertise using high-performance wireless W-LAN networks. In addition, the application (airRX) would provide physicians on board with insight into various scenarios and, thus, an overview of medical equipment.

The number of air passengers is increasing worldwide. Even though the SARS-CoV-2/COVID-19 pandemic led to a temporary reduction of over 50% in global passenger numbers in 2020 and 2021, the number has evolved from approximately 1 billion annual passengers in 2004 to more than four billion/year by 2019 [22]. However, the airline industry has not yet recovered from the pandemic situation. Expectations and future projections should be highly debated now, considering rising energy prices, low emissions demanded by policy makers in the face of challenges with lower CO^2^ or pollutant emissions, keywords such as climate change and ecological footprint, and demanded “green” and modern technologies. The largest German airline with international operations, Lufthansa AG, and its subsidiaries, Austrian Airlines AG (Vienna, Austria) and SWISS (Swiss International Air Lines Ltd., Zurich, Switzerland), launched the “Arzt an Bord/Doctor on Board” program many years ago [18]. Physicians, dentists, and specialists from all fields can register with this program to be able to offer rapid assistance in the event of a medical emergency. In doing so, the crew of the aircraft can easily and discreetly identify doctors on the flight by identifying them on the passenger list. Doctors are offered benefits if they choose to participate in the program, such as a one-time credit of Miles & More miles. The Miles & More frequent flyer program is a company bonus/credit system with a status system with possible privileges, where air miles are earned to get flights cheaper or even for free as gratitude from the company or as a benefit for loyalty or customer retention. Various privileges of the participant include, for example, baggage tags, priority or fast check-in, access to various lounges with the possibility of eating, drinking, resting, lying down, showering, going to the toilet, or business infrastructure, such as WIFI, telephone, smartphone, and laptop chargers, as well as power outlets. Above all, services such as an increased baggage allowance or waiting list priority, as well as exclusive service telephone hotlines, are amenities popular with frequent flyers, making frequent business travel easier. In the case of medical assistance provided by onboard physicians, additional gratuities are also provided. As participants in the “Doctors on Board” program, physicians are also covered by special liability insurance when treating patients on board the aircraft [18].

### 5.2. Use of Telemedicine in Train Traffic and Shipping

Emergencies in train traffic should not go unmentioned in this context. Especially in long-distance trains, it has been revealed that rarely, very weak reception or a greatly reduced bandwidth, if any, is available. For emergencies, which are not uncommon even among long-distance travelers, the possibility of a telemedical emergency presentation with first aid measures until the arrival of the rescue service or medical assistance is rather difficult to ensure, so that the early connection of an emergency physician via telemedicine may be technically difficult or even impossible, and a primary assessment of the situation can only be transmitted by lay helpers by telephone. Emergency situations can also occur, for example, on a cruise or even far away from the mainland on the high seas. Even though there are often medical professionals on board, further diagnostics by specialists may be necessary. For example, cooperation between the cruise ship company AIDA Cruises and the University Medical Center Rostock has been established to transfer specialized university knowledge to any ship of the AIDA fleet in case of need. The so-called on-board hospitals of the AIDA ships allow imaging examination procedures, such as X-rays or even ultrasound examinations on the high seas. Through the pioneering project of the two mentioned cooperation partners, specialist medical expertise of the university medicine was made available for further examination or evaluation of the data by means of telemedical applications in the field of radiology via satellite connection [23].

### 5.3. Teledentistry in Dental Disciplines

Options for telemedical applications offer simplified access to rural areas and thus bridge dental care in sparsely populated areas without the establishment of a dentist. In addition, factors include time savings for the patient to obtain an expert’s opinion and a low-cost initial consultation. Advantages are also gained by improving interpersonal communication, such as obtaining second opinions or inquiring about cost approvals from health insurers or payers. Although it is widely used in medicine, teleradiology as an essential part of telemedicine until now has not really been established in dentistry [24]. Given the huge number of dental radiographs acquired in the world each year, this potential has yet to be explored. The introduction of a teleradiology system in general dental practice could be helpful for the differential diagnosis of oral lesions and the reduction of costs [24]. Telemedicine can also offer solutions in various dental disciplines, such as endodontics, e.g., by using images after a prepared access cavity (after trepanation/opening of the tooth for root canal treatment) to identify the pulp chamber floor and thus root canal entrances [25]. There are also options for assessing orthodontic issues, such as the severity of malocclusions in the teeth or jaws [26]. Teledental applications in prosthodontics may offer the possibility, especially for diagnostics and planning for the rehabilitation of function in the field of gerodontology, to consult immobile patients in nursing homes and to obtain information about treatment needs by means of presentation via video consultation [27]. However, it has also been demonstrated in the dental disciplines of pediatric dentistry, preventive dentistry, and periodontics to provide access to treatment for underprivileged populations [28,29]. Its use in dental traumatology has also been shown to provide valuable support to patients when no dentist is immediately available or accessible [30]. A digital workflow of various techniques has not only changed or improved through CAD-CAM procedures but has enabled digital planning of entire jaw reconstructions in oral and maxillofacial surgery, as well as fully digital planning for the rehabilitation of masticatory function based on implants. These advances in the use of computers, telecommunication technology, and digital diagnostic imaging techniques have had a significant positive impact on dental services through the development of new equipment and software for analysis and follow-up treatments [31]. Remote diagnostic applications, such as in the diagnosis of oral lesions and other pathologies via the transmission of images by e-mail, can be accelerated. Thus, potentially costly, time-consuming, and often difficult patient transport may also render treatment redundant in its efficiency and effectiveness when preoperative assessment is evaluated [32].

### 5.4. Telemedicine Applications in Urban and Rural Areas

In addition to the use of telemedicine applications in university hospitals or academic medical centers, applications are nowadays also used in community hospitals in both urban and rural areas. Nationally and internationally, the number of vendors or service providers working on this topic is increasing. The linking of developing countries with hospitals in industrialized countries should be mentioned at this point. For many years, health insurance companies in Switzerland have offered the option of a discounted rate, whereby a specialist is first contacted telemedically about the corresponding patient case, and then a recommendation is made for further treatment [33].

### 5.5. Telemedicine Applications in International Aid Projects

Working groups around the world used telemedical applications through their aid projects to improve (oral) health in their respective countries. Due to the SARS-CoV-2/COVID-19 pandemic, the planned visits with training and calibration of dental colleagues or dental public health officers, teachers, parents, and children could not be carried out on site. As an alternative, to still make the implementation as possible as possible, the digital possibilities were exhausted, and online training occurred, some of which were recorded or live-streamed. This challenge was only possible with the help of advances in digital communication or telecommunications via the internet, which conditionally also enabled the possibility of remote access to medical care or at least education and information [34]. Several aid projects linked to local hospitals have made it possible to add dental concerns to general medical services to improve oral health.

### 5.6. Telemedicine Application in Continuing Dental/Medical Education

It is possible to divide formal online education into two areas: web-based self-instruction and interactive videoconferencing. The advantages of web-based self-learning are the self-determined pace of learning and the possibility of repetition as often as desired. Disadvantages include difficulty ascertaining reflection on learning and, consequently, a lack of accuracy and deficits in terms of learner satisfaction [35]. Lack of communication is often described as one of the main reasons for dissatisfaction [36]. Interactive videoconferencing, e.g., via satellite or internet or intranet, includes both live interactive videoconferencing and supporting information such as the patient’s medical history and findings such as radiographs. An exchange in teaching between, e.g., instructor and student, if necessary, with or without the presence of the patient allows interaction and feedback, and the learning pace can be adjusted individually [34]. However, it has already been observed that e-mail-based consultations in dentistry reveal that face-to-face patient examinations allow for a more correct diagnosis of oral mucosal diseases than the mere transmission of descriptive patient data [37].

## 6. Obstacles of Telemedicine and Teledentistry

Problems that have still not been solved include the lack of telecommunication interfaces, the interconnection of the practice and thus the connection to a network, and, above all, the data protection situation. Unfortunately, all traffic via the internet may be subject to fraudulent external access, even when all security measures are adopted. While this general drawback of modern digital data exchange networks cannot be generally avoided yet, changes in the Patient Data Protection Act are intended to remedy the legal data-protection situation and pave the way for digital applications that can facilitate everyday practice. While no certificates of incapacity to work or medications may be prescribed for unknown patients via video chat to date [15], remote treatment is also considered a popular option among psychotherapists in times of pandemic, albeit with special attention and extraordinary duties of care. Exclusive remote treatment is rejected by most physicians on the grounds that in-depth diagnostics always include face-to-face conversations with physical presence. An impression of the patient only via video has clear disadvantages; a handshake, which was still common before the pandemic, the smell, as well as the physiological changes in the personal conversation, have proven to be useful and provided additional information. For example, a possible ketosis (metabolic state with an increase in the concentration of acidic ketone bodies), as in insufficiently controlled diabetes mellitus or also a ketoacidosis (complication of absolute insulin deficiency), could be perceived, and a life-threatening coma avoided at an early stage. Cold sweating (profuse sweating with cold skin), which usually occurs in stressful situations and indicates a severe clinical picture, such as shock, myocardial infarction, or pulmonary edema, as an accompanying symptom, is also easier to recognize if the patient is presented to the physician in person. By presenting the patient via video, the quality of the transmission also depends on the hardware and software, as well as the bandwidth and the video software used by the two interlocutors, the doctor, and the patient. Access should be granted under no circumstances only to people who have the financial means to buy an up-to-date internet-capable smartphone with the necessary mobile phone contract or a sufficiently covered prepaid card. The software required for a video call must be designed in such a way that it is easy to use without much effort, requires little memory, and can be used compatibly on all common operating systems of both mobile devices and computer systems (e.g., Microsoft, Apple, and Linux). Legislation should also be reviewed so that access for all people can be guaranteed, as was the case with emergency pillars on the highway or the yellow telephone booths in Germany. Other challenges in this context include the need for regular updates of device software and the provision of sufficient network coverage in both urban and rural areas [38].

### 6.1. Problems of Telemedicine in Dental Education, Training, and Continuing Education

Although telemedicine provides promising options in the field of dental education and training, its limitations and critical factors must also be mentioned. In this context, legal questions or legal issues are particularly important to consider. These include, for example, data protection, ethical aspects, safety, licensing, and malpractice. Pedagogical issues, such as sustainability, standards, selection of instructors, or protocol design, can also present limitations. Due to state licensing conditions and requirements, even dental continuing education courses, such as X-ray protection updates, may be prohibited by law or may have requirements, such as requiring a camera to be turned on and the face to be shown throughout the entire continuing education course.

### 6.2. Challenges, Difficulties, and Risks of Using Telemedicine

Although access to dental care can be improved, applications across state borders may be difficult [39]. Although the European Single Market allows cross-national services for the most part, there may be problems with billing, for example. If technical problems arise during data transmission that then result in medical errors or even misdiagnoses, the question of liability is often not easy to answer [40]. Privacy and security also pose obstacles, especially when patient data are either lost, stolen, or even manipulated [35]. However, there are more than just solutions being discussed on the part of the medical profession. Concerns and fears are also expressed by physicians that they may be replaced by a call center in the future, if necessary, or that the quality of care would deteriorate. The very important relationship between doctor and patient could also deteriorate because of digital distance, although it is already known that personal contact is also essential for the patient’s healing. Dentists also fear the use of telemedical applications to some extent, such as in the use of aligners (transparent plastic splints for orthodontic therapy or correction of malpositioned teeth) [41]. These are offered by various dental networks or chains. After initial assessment and diagnosis by a dentist, 24 prefabricated splints, for example, are then sent to the patient over a period of two years. However, it is not only the therapy and the result that can be jeopardized by such applications. Personal doctor–patient contact is also lost, and freelancing can be jeopardized, for example, by investor-driven and purely profit-oriented companies not respecting and considering the characteristics of freelancing, which can significantly change the dental care landscape in the future. The characteristic feature of the liberal profession, in addition to the commitment to the common good of the independently and personally provided service, is the economically and professionally independent performance of tasks. This goes hand in hand with a special relationship of trust between doctor and patient and the postponement of the interest in maximizing profits [42].

## 7. Conclusions

Telemedicine and teledentistry have a wide range of applications. Today, they already offer the possibility of being used in many places for diagnostics, therapy, rehabilitation, and decision-making consultations across spatial distances. Technical and semantic standards are required for smooth handling and must still be defined in various areas. The experience gained from video and online emergency consultations during the pandemic should be used and expanded so that patients can benefit from it worldwide. Legal and privacy hurdles often still need to be resolved, posing challenges for users as well as software and hardware manufacturers. Obstacles in internet coverage must also be solved in rural areas to enable access; socially disadvantaged and vulnerable groups should be supported in both technology and handling. Even though, especially in dentistry, many medical specialties still must be performed clinically through hands-on/manual examinations, there are areas of diagnostics where telemedicine applications can provide good support. Telemedicine and teledentistry applications should be provided, expanded, and improved to enable access for all population groups. However, based on this narrative review, a systematic review focusing on the individual areas mentioned above should be conducted.

## Data Availability

Not applicable.

## References

[1] German Medical Association (Bundesärztekammer) (2015). Telemedizin. https://www.bundesaerztekammer.de/fileadmin/user_upload/_old-files/downloads/pdf-Ordner/Telemedizin_Telematik/Telemedizin/V-03_Entschliessung_Telemedizin.pdf.

[2] World Health Organizsation (WHO) (2005). 58. World Health Assembly. WHA58.28 eHealth. http://apps.who.int/iris/bitstream/10665/20378/1/WHA58_28-en.pdf.

[3] Working Group Telemedicine of the German Medical Association (Bundesärztekammer) (2015). Telemedizinische Methoden in der Patientenversorgung–Begriffliche Verortung. https://www.bundesaerztekammer.de/fileadmin/user_upload/_old-files/downloads/pdf-Ordner/Telemedizin_Telematik/Telemedizin/Telemedizinische_Methoden_in_der_Patientenversorgung_Begriffliche_Verortung.pdf.

[4] Thrall J.H. (2007). Teleradiology. Part I. History and clinical applications. Radiology.

[5] American College of Radiology (ACR) (1994). Standards for Teleradadiology.

[6] VeSta Informationsportal. https://www.informationsportal.VeSta-gematik.de/ueber-VeSta/.

[7] Ericsson (2015). Ericsson Mobility Report–On the Pulse of the Networked Society. https://www.ericsson.com/49df10/assets/local/reports-papers/mobility-report/documents/2015/ericsson-mobility-report-june-2015.pdf.

[8] Nier H. (2018). Mobiles Internet–Die Schnellsten LTE-Netze Europas. https://de.statista.com/infografik/12986/schnellste-lte-netze-europas/.

[9] Robert Koch Institute (RKI) (2022). COVID-19 in Germany. https://www.rki.de/EN/Home/homepage_node.html.

[10] Wolf T.G., Deschner J., Schrader H., Bührens P., Kaps-Richter G., Cagetti M.G., Campus G. (2021). Dental Workload Reduction during First SARS-CoV-2/COVID-19 Lockdown in Germany: A Cross-Sectional Survey. Int. J. Environ. Res. Public Health.

[11] Cagetti M.G., Balian A., Camoni N., Campus G. (2021). Influence of the COVID-19 Pandemic on Dental Emergency Admissions in an Urgent Dental Care Service in North Italy. Int. J. Environ. Res. Public Health.

[12] COVIDental Collaboration Group (2021). The COVID-19 pandemic and its global effects on dental practice. An international survey. J. Dent..

[13] Kassenzahnärztliche Bundesvereinigung und Bundeszahnärztekammer (2020). Maßnahmen der Zahnärzteschaft für Die Aufrechterhaltung der Versorgung. https://www.bzaek.de/berufsausuebung/sars-cov-2covid-19/massnahmenpaket-der-zahnaerzteschaft.html.

[14] Schubert J., Nokaj M. (2020). Patientenkommunikation. Telemedizin: Auch für Zahnärzte Sinnvoll. https://www.quintessence-publishing.com/deu/de/news/praxis/patientenkommunikation/telemedizin-auch-fuer-zahnaerzte-sinnvoll.

[15] KV FUX (2019). https://www.kv-fux.de/2019/03/19/fernbehandlungsverbot/.

[16] International Air Transport Association (IATA) (2021). Annual Review 2021. https://www.iata.org/contentassets/c81222d96c9a4e0bb4ff6ced0126f0bb/iata-annual-review-2021.pdf.

[17] International Air Transport Association (IATA) (2022). World Air Transport Statistics (WAtS). https://www.iata.org/en/publications/store/world-air-transport-statistics/.

[18] Lufthansa (2022). Arzt an Bord–News. https://www.lufthansa.com/et/de/arzt-an-bord-news.

[19] Siedenburg J. (2005). Kompendium Flug- und Reisemedizin.

[20] Siedenburg J., Küpper T. (2015). Moderne Flugmedizin.

[21] Graf J., Stüben U., Pump S. (2012). Medizinische Notfallsituationen im Flugzeug. Dtsch. Arztebl. Int..

[22] Mazareanu E. (2021). Global Air Traffic–Scheduled Passengers 2004–2022. https://www.statista.com/statistics/564717/airline-industry-passenger-traffic-globally/.

[23] Operation Karriere (2016). Von Beruf Arzt. Telemedizin: Kooperation Optimiert Versorgung auf AIDA-Schiffe. https://www.operation-karriere.de/karriereweg/von-beruf-arzt/telemedizin-optimiert-versorgung-auf-aida-schiffen.html.

[24] Choi J.W. (2013). Clinical usefulness of teleradiology in general dental practice. Imaging Sci. Dent..

[25] Brüllmann D., Schmidtmann I., Warzecha K., d’Hoedt B. (2011). Recognition of root canal orifices at a distance–a preliminary study of teledentistry. J. Telemed. Telecare.

[26] Park J.H., Kim J.H., Rogowski L., Al Shami S., Howell S.E.I. (2021). Implementation of teledentistry for orthodontic practices. J. World Fed. Orthod..

[27] Ignatius E., Perälä S., Mäkelä K. (2010). Use of videoconferencing for consultation in dental prosthetics and oral rehabilitation. J. Telemed. Telecare.

[28] Kopycka-Kedzierawski D.T., Billings R.J. (2006). Teledentistry in inner-city child-care centres. J. Telemed. Telecare.

[29] Jampani N.D., Nutalapati R., Dontula B.S., Boyapati R. (2011). Applications of teledentistry: A literature review and update. J. Int. Soc. Prev. Community Dent..

[30] Lienert N., Zitzmann N.C., Filippi A., Weiger R., Krastl G. (2010). Teledental Consultations Related to Trauma in a Swiss Telemedical Center-A Retrospective Survey. Dent. Traumatol..

[31] Clark G.T. (2000). Teledentistry: What is it Now, and What Will it be Tomorrow?. J. Calif. Dent. Assoc..

[32] Ibraheim A., Sanalla A., Eyeson J. (2021). The role of teledentistry in oral surgery during the COVID-19 pandemic. Adv. Oral Maxillofac. Surg..

[33] Diener E. Medinside: Neue Krankenversicherung: Zuerst zur App statt zum Arzt. https://www.medinside.ch/post/neue-krankenversicherung-zuerst-zur-app-statt-zum-arzt.

[34] Dils E.S., Lefebvre C., Abeyta K. (2004). Teledentistry in the United States: A New Horizon of Dental Care. Int. J. Dent. Hyg..

[35] Chen J.W., Hobdell M.H., Dunn K., Johnson K.A., Zhang J. (2003). Teledentistry and its use in dental education. Am. Dent. Assoc..

[36] Spallek H., Pilcher E., Lee J.Y., Schleyer T. (2002). Evaluation of Web-based dental CE course service. J. Dent. Educ..

[37] Younai F.S., Messadi D.V. (2002). E-mail-based oral medicine consultation. J. Calif. Dent. Assoc..

[38] Logitel (2018). Netzabdeckung in Deutschland–der Große Überblick!. https://www.logitel.de/blog/handys/netzabdeckung-in-deutschland-der-grosse-ueberblick/.

[39] Golder D.T., Brennan K.A. (2000). Practicing dentistry in the age of telemedicine. JADA.

[40] Biegel S. (2000). Virtual Health Care: Unresolved Legal Issues. J. Calif. Dent. Assoc..

[41] Olk J. (2021). Zahnschienen. Koalition will Tele-Zahnmedizin Schärfer Regulieren. https://www.handelsblatt.com/inside/digital_health/zahnschienen-koalition-will-tele-zahnmedizin-schaerfer-regulieren/27206608.html?ticket=ST-13107287-xd0fQyNhCmksGTMegixz-ap1.

[42] Europäischer Wirtschafts- und Sozialausschuss (EWSA) (2013). Die Lage der Freien Berufe in Ihrer Funktion und Bedeutung für die Europäische Zivilgesellschaft. Zusammenfassung. EESC/COMM/05/2013. https://www.eesc.europa.eu/sites/default/files/resources/docs/qe-01-13-678-de-c.pdf.

